# Effectiveness of a Family Education Intervention Using an AI-Supported Video in Postoperative Care of Children with Cleft Lip and Palate: A Pilot Pre–Post Study

**DOI:** 10.3390/healthcare14142182

**Published:** 2026-07-20

**Authors:** Şükran Öztürk, Sermin Dinç

**Affiliations:** 1 Şişli Etfal Eğitim ve Araştırma Hastanesi, Istanbul 34453, Türkiye; 2 Pediatric Nursing Department, Faculty of Health Sciences, İstanbul Atlas University, Istanbul 34408, Türkiye

**Keywords:** cleft lip and palate, artificial intelligence, video-supported education, postoperative care, mother education

## Abstract

**Highlights:**

**What are the main findings?**

- This pilot study suggests that AI-supported video-based education may help mothers better understand how to care for their child after cleft lip and/or palate surgery.
- The biggest gain was in recognizing and monitoring complications, which suggests this approach could help close an important gap in mothers’ postoperative care knowledge.

**What are the implications of the main findings?**

- AI-supported video-based education may be a practical and supportive way to strengthen family-centered postoperative education in pediatric surgical care.
- Larger, well-controlled studies with longer follow-up are still needed to confirm these early findings and better understand how they affect caregiving practices over time.

**Abstract:**

**Background/Objectives:** Cleft lip and palate are among the most common congenital craniofacial anomalies and require careful postoperative care after surgical repair. Mothers, as primary caregivers, are expected to manage feeding, wound care, oral hygiene, and the early recognition of complications; however, gaps in postoperative care knowledge may limit safe home care. This pilot study aimed to evaluate the effect of an artificial intelligence (AI)-supported video-based educational intervention on mothers’ knowledge of postoperative care after cleft lip and/or palate surgery. **Methods:** This single-group quasi-experimental pre–post pilot study was conducted between April and December 2025 in the Plastic, Reconstructive, and Aesthetic Surgery Clinic of a tertiary hospital in Istanbul. Thirty mothers of children aged 0–18 years who underwent cleft lip and/or palate surgery were included. Data were collected using a Demographic Information Form and a Nutrition and Care Knowledge Questionnaire. Mothers completed the questionnaire before and immediately after watching a standardized AI-supported educational video developed by the researchers. Pre- and post-intervention knowledge scores were compared using nonparametric statistical tests. **Results:** A total of 30 mothers participated in the study. Child-related sociodemographic and clinical variables were recorded to describe the children’s profile. Among the children, 56.7% were female, and 56.7% were aged 0–3 years. Post-intervention knowledge scores increased in the total score and in all subdomains, including postoperative care, feeding, and complication monitoring. The greatest improvement was observed in complication monitoring, which had the lowest baseline scores. Knowledge gains were observed across participant subgroups. **Conclusions:** This pilot study suggests that AI-supported video-based education may improve mothers’ short-term knowledge of postoperative care after cleft lip and/or palate surgery. However, because of the single-group design, small sample size, and immediate post-test assessment, the findings should be interpreted as preliminary. Larger controlled studies with longer follow-up are needed to examine knowledge retention and the potential effects of this approach on caregiving practices.

## 1. Introduction

Cleft lip and palate rank among the most prevalent congenital craniofacial anomalies worldwide and represent a considerable burden for affected children and their families alike [1,2]. Beyond their visible impact on facial aesthetics, these malformations interfere with a range of essential functions—feeding, speech, hearing, dental development, and psychosocial adaptation—across different stages of childhood [3,4]. In early life, particularly, persistent feeding difficulties can compromise growth trajectories and nutritional status, with downstream consequences for overall health and quality of life [5,6,7].

Surgical repair remains the cornerstone of treatment, yet the operative procedure itself addresses only one dimension of a far more complex care continuum. The postoperative period carries its own critical demands: wound care, oral hygiene, infection surveillance, early detection of complications, and—perhaps most consequentially—appropriate feeding management. In clinical practice, the responsibility for all of these tasks falls predominantly on mothers [8,9]. Yet the literature consistently documents a striking gap between what mothers are expected to know and what they actually know, with deficits in feeding-related knowledge appearing particularly pronounced [9,10,11].

Conventional approaches to patient and family education—verbal instruction, brief counselling sessions, printed materials—have well-recognized limitations. Information delivered in a single encounter is easily forgotten, difficult to revisit, and poorly suited to the heightened stress and anxiety that characterize the immediate postoperative environment. Under such conditions, even motivated caregivers may struggle to absorb, retain, and apply what they have been taught [9,10].

Against this backdrop, the integration of digital technologies and artificial intelligence into health education has attracted growing interest [12,13,14,15]. AI-supported modalities allow learners to engage with content at their own pace, to revisit material as many times as necessary, and to benefit from audiovisual formats that have been shown to support the comprehension of complex procedural information [12,13]. Video-based educational resources, in particular, have demonstrated promise in making intricate care tasks more accessible and in fostering greater caregiver engagement [9]. Within pediatric settings, where mothers occupy a central and often irreplaceable role in the care process, raising the level of maternal knowledge is not merely desirable—it is a prerequisite for safe, consistent, and effective postoperative management [3,4,8].

Despite the expanding use of AI-supported digital tools in clinical education more broadly, evidence regarding their application in the specific context of maternal education following cleft lip and/or palate surgery remains sparse [12,13,14,15]. AI-produced video-based materials offer a potentially distinctive contribution here: their standardized, reproducible structure reduces variability in content delivery; their audiovisual components may strengthen knowledge encoding and retention; and their accessibility after discharge allows mothers to return to key information precisely when they need it most. Collectively, these features may also support parental self-efficacy and help reduce the incidence of inappropriate caregiving practices that predispose to postoperative complications [8,9,12,13,14,15].

The present study was therefore designed to evaluate the effect of an artificial intelligence-assisted educational video—developed specifically for mothers of children aged 0–18 years who had undergone cleft lip and/or palate surgery—on maternal knowledge of postoperative care.

Hypothesis:

**H0.**  *There will be no statistically significant difference between pre-intervention and post-intervention knowledge levels of mothers who receive AI-supported video-based education.*

**H1.**  *Mothers who receive the AI-supported video-based education will demonstrate a statistically significant increase in knowledge levels after the intervention compared to before the intervention.*

## 2. Materials and Methods

### 2.1. Study Design

**Figure 1:**
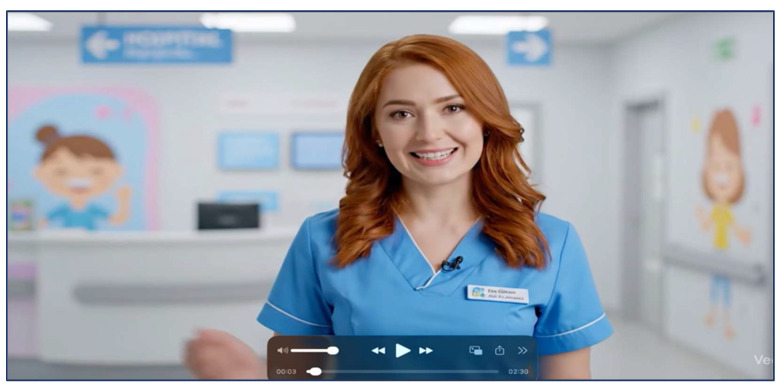
AI-supported video-based education video for cleft lip and/or palate care.

**Figure 2:**
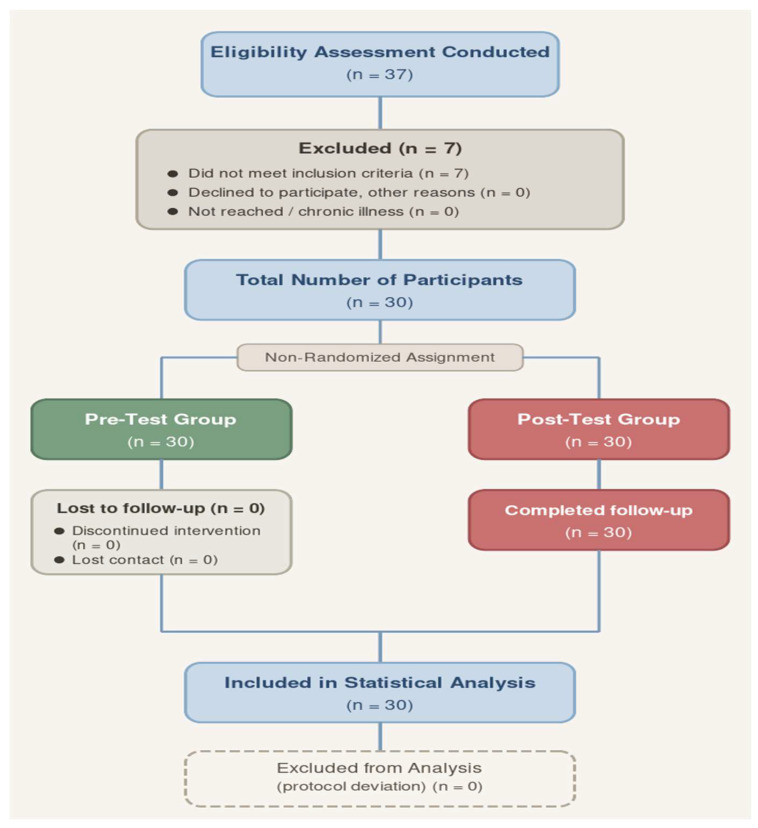
Participant flow diagram according to the TREND guideline.

This study was designed as a single-group pre–post quasi-experimental study to evaluate the effect of an artificial intelligence-assisted educational video (developed using AI-based and AI-assisted tools, including Google Veo 3/3.1, Midjourney v6.0/v6.1, CapCut v4.0+, and Opus Clip/Veed.io) (Figure 1) on the knowledge levels of mothers of children aged 0–18 years who had undergone cleft lip and/or palate surgery, and can be considered a pilot study providing preliminary evidence. The study was reported in accordance with the TREND (Transparent Reporting of Evaluations with Nonrandomized Designs) guidelines (Figure 2; Supplementary File S2).

### 2.2. Setting and Participants

This study was conducted between April and December 2025 in the Plastic, Reconstructive, and Aesthetic Surgery Clinic of a Training and Research Hospital in Istanbul. The study participants were mothers of children aged 0–18 years who underwent cleft lip and/or palate surgery. Mothers were included because they were the primary caregivers responsible for the postoperative care process. Child-related variables, including age, sex, diagnosis type, and surgical procedure, were collected only to describe the clinical and surgical profile of the children. The sample included all eligible mothers admitted to the clinic during the specified period and who met the inclusion criteria. During the study period, 37 mothers of children who underwent cleft lip and/or palate surgery were assessed for eligibility. Seven mothers were excluded because they did not meet the inclusion criteria or declined to participate. Therefore, 30 eligible mothers were recruited into the study. All recruited mothers completed the pre-test, received the AI-supported video-based education intervention, completed the post-test, and were included in the final analysis (n = 30).

#### Sample Size Justification

Given the pilot nature of the study, the sample size was determined pragmatically based on participant availability and recruitment feasibility during the predefined data collection period. No formal a priori power analysis was conducted, as the study was not designed to provide definitive evidence of effectiveness. Rather, it aimed to generate preliminary evidence regarding the feasibility of the AI-supported video-based educational intervention and short-term changes in mothers’ knowledge. Accordingly, the inclusion of 30 mothers was considered acceptable for a pilot pre–post study and useful for informing the design, sample size estimation, and outcome selection of future controlled trials.

Inclusion Criteria:

- Having a child who underwent cleft lip and/or palate surgery.
- Actively participating in the postoperative care process.
- Being able to read and understand Turkish.
- Providing voluntary consent to participate in the study.

Exclusion Criteria:

- Mothers with developmental or cognitive impairments.
- Individuals without sufficient technological access to receive video-based education.
- Mothers who had previously received training on the same topic.

### 2.3. Data Collection

This study used a single-group pretest–posttest quasi-experimental design with mothers of children who underwent cleft lip and/or palate surgery.

Mothers to be included in the study were selected from patients evaluated in the postoperative pediatric ward. Written and verbal informed consent was obtained from eligible mothers who agreed to participate.

The data collection process was initiated approximately one hour after admission to the postoperative ward. At this stage, the Demographic Information Form was administered to the mothers, and their sociodemographic characteristics and caregiving experiences were recorded. To determine baseline knowledge levels, the Nutrition and Care Knowledge Questionnaire was administered as a pretest.

Subsequently, mothers were shown an artificial intelligence-supported educational video developed within the scope of the study. The video included information on cleft lip and palate, the surgical process, postoperative care, nutrition, and possible complications. The training was delivered to all participants under standardized conditions, in the same environment and using the same device, for approximately 15 min.

After the training, the Nutrition and Care Knowledge Questionnaire was re-administered as a post-test, allowing for comparison of mothers’ knowledge levels before and after the intervention. The post-test was administered on the same day as the pre-test, immediately after the mothers watched the AI-supported video-based educational material. Therefore, the study evaluated short-term changes in mother knowledge rather than long-term knowledge retention. After completion of the post-test, the educational video was shared with the mothers as a digital video link/file via WhatsApp so that they could rewatch it when needed during the postoperative home care process. The researcher’s contact information was also provided for any potential questions.

Demographic Information Form:

The form, developed by the researcher in line with the relevant literature to determine the descriptive characteristics of children who underwent cleft lip and/or palate (CLP) surgery, consists of 9 questions. It includes information about both mothers and children, such as age, gender, weight, and educational level.

Nutrition and Care Knowledge Questionnaire:

The Nutrition and Care Knowledge Questionnaire was developed by the researchers to assess mothers’ postoperative care knowledge after cleft lip and/or palate surgery. The initial item pool was prepared based on the relevant literature, clinical recommendations, and the educational content of the intervention. The questionnaire consisted of 15 dichotomous items grouped into three domains: general postoperative care knowledge, feeding knowledge, and complication monitoring and long-term follow-up knowledge.

To support content validity, the questionnaire was reviewed by three experts: one plastic surgeon and two specialist physicians. The experts evaluated each item in terms of relevance to the study objective, clarity, scope adequacy, and clinical appropriateness. Items were revised according to expert feedback, and the final version of the questionnaire was established.

Internal consistency was examined using Cronbach’s alpha. For the pre-test, the Cronbach’s alpha coefficient was 0.866 for the total questionnaire, indicating good internal consistency. The alpha coefficients for the subdomains were 0.893 for postoperative care knowledge, 0.733 for feeding knowledge, and 0.869 for complication monitoring knowledge. Corrected item–total correlations ranged from 0.271 to 0.835, suggesting acceptable item discrimination. For the post-test, the Cronbach’s alpha coefficient was 0.672. The lower post-test alpha may reflect restricted variance caused by clustering of correct responses after the intervention, as items Q01–Q09 showed no variance because all mothers answered these items correctly.

Because this was a pilot study with a limited sample size, comprehensive psychometric testing, including construct validity analysis and test–retest reliability, was not performed. Therefore, the questionnaire should be considered a researcher-developed knowledge assessment tool, and the related findings should be interpreted as preliminary.

The questionnaire consists of 15 items prepared to assess postoperative nutrition and care knowledge of mothers:

- General Postoperative Care Knowledge: 5 items.
- Nutrition Knowledge: 5 items.
- Complications and Long-Term Follow-Up Knowledge: 5 items.

Scoring Recommendation:

- “Yes” response to a correct statement = 1 point.
- “Yes” to an incorrect statement or “Not sure” = 0 points.

Total score range: 0–15.

Score interpretation:

- 0–5 = Low level of knowledge.
- 6–10 = Moderate level of knowledge.
- 11–15 = High level of knowledge.

AI-supported video-based education for cleft lip and/or palate care

The educational video was developed by the researcher, who received formal training in artificial intelligence (AI)-assisted video production as part of a master’s thesis. Multiple AI-based tools were utilized throughout the development process, including avatar generation, visual content design, and automated voice synthesis.

In this study, the term “AI-assisted” refers specifically to the use of artificial intelligence-based tools during the production phase of the educational video. AI-supported applications were used for avatar generation, automated voice synthesis, and visual content design. The educational content was prepared by the researchers in accordance with current clinical recommendations and relevant literature, and its clinical accuracy was reviewed by healthcare professionals. The AI component did not provide adaptive learning, personalized feedback, real-time interaction, parent-specific recommendations, or clinical decision support during educational delivery. Therefore, the intervention should be interpreted as a standardized AI-supported video-based education material rather than an interactive AI-driven educational system.

The final video was delivered under standardized conditions, ensuring that all participants received the same educational content in a consistent format. In addition, the video was made accessible in digital form to support repeated viewing and reinforce learning after discharge (Supplementary File S1).

### 2.4. Sociodemographic and Clinical Variables

To characterize the study sample, sociodemographic data of the mothers, including age, educational level, socioeconomic status, and caregiving experience, were collected. In addition, child-related variables, including age, gender, diagnosis type, and surgical procedure characteristics, were recorded.

Mothers’ prior exposure to information on similar caregiving processes, their experience receiving education from healthcare professionals, and their experiences with postoperative care practices were also assessed. Furthermore, the length of hospital stay and additional information related to the caregiving process were included in the data collection.

### 2.5. Data Analysis

In this study, changes in mothers’ knowledge levels regarding the care of children who underwent cleft lip and/or palate surgery were evaluated over time using pre-test and post-test measurements. Since repeated measurements were obtained from the same individuals, the Wilcoxon Signed-Rank Test was used to analyze differences between paired observations. The predominance of negative ranks in the test statistics indicated that post-test scores were higher than pre-test scores, reflecting an improvement in knowledge levels.

Statistical analyses were performed using IBM SPSS Statistics (version 31). Descriptive statistics were presented as mean ± standard deviation, median (interquartile range [IQR]), minimum, and maximum for continuous variables, and as frequencies and percentages for categorical variables.

Given the data’s distribution characteristics and the limited sample size, nonparametric statistical methods were preferred. Differences between pre-test and post-test measurements were analyzed using the Wilcoxon Signed-Rank Test for paired data. In addition to statistical significance, effect size was calculated using the formula r = |Z|/√N.

To examine changes in knowledge levels in greater detail, delta scores were calculated for each subdimension and the total knowledge score by subtracting pre-test scores from post-test scores. These change scores were compared across binary variables (e.g., gender and diagnosis type) using the Mann–Whitney U test, and across variables with more than two categories (e.g., age groups and parental education level) using the Kruskal–Wallis test.

To determine which domains of knowledge showed greater improvement following the educational intervention, delta values for postoperative care knowledge, feeding knowledge, complication monitoring knowledge, and total knowledge scores were compared using the Friedman test. The magnitude of differences and the level of agreement among knowledge domains were evaluated using Kendall’s coefficient of concordance (W).

Relationships between clinical and educational variables were analyzed using Spearman’s rank correlation coefficient (Spearman’s rho), as the variables were ordinal and did not meet the assumption of normal distribution. Specifically, associations between length of hospital stay and knowledge change scores, as well as between parental education level and delta scores, were examined.

A *p*-value of < 0.05 was considered statistically significant. Given the small sample size and multiple comparisons, the findings were interpreted not only in terms of statistical significance but also with consideration of clinical and educational relevance.

### 2.6. Ethical Consideration

Ethical approval for the study was obtained from the Non-Interventional Clinical Research Ethics Committee of Atlas University (Approval No: E-22686390-050.99-6489; Decision No: 04/33; Date: 28 April 2025). Permission to conduct the study was also granted by the Research, Publications, and Announcement Content Evaluation Commission of the Provincial Directorate of Health (Decision No: 2025-13; Date: 30 October 2025).

Institutional approval was obtained from the Education Planning Committee of Istanbul Şişli Hamidiye Etfal Training and Research Hospital (Approval No: E-79.341.859-604.01-277462954). Before data collection, the study’s purpose was explained to the children’s legal guardians, and written informed consent was obtained.

## 3. Results

### 3.1. Sample Characteristics

A total of 30 mothers of children who underwent surgical intervention for cleft lip and/or palate were included in the study. The sociodemographic characteristics of the mothers and children are presented in Table 1. Child-related variables were reported to describe the clinical profile of the children. Among the children, 56.7% were female, and 56.7% were in the 0–3 years age group. The majority of mothers had completed high school, while 23.3% had completed primary education.

The distribution of the children’s clinical characteristics in the study is presented in Table 2. Regarding diagnosis types, 50.0% of the children had cleft lip, 46.7% had cleft palate, and 3.3% had both cleft lip and palate.

Regarding surgical procedures, 46.7% of the children underwent cleft lip surgery, 46.7% underwent cleft palate surgery, and 6.7% underwent surgery for both cleft lip and palate. None of the children had a history of chronic disease or a family history of similar conditions.

### 3.2. Internal Consistency of the Knowledge Questionnaire

The pre-test internal consistency of the questionnaire was good, with a Cronbach’s alpha coefficient of 0.866 for the total scale. Subdomain alpha coefficients ranged from 0.733 to 0.893. The post-test alpha coefficient was 0.672, which may reflect restricted variance due to the clustering of correct responses after the intervention.

Table 3 presents the changes in knowledge levels regarding the care of children who underwent cleft lip and/or palate surgery over time. Post-test scores were markedly higher than pre-test scores across all subdimensions and in the total knowledge score. Descriptive statistics showed a significant increase in both mean and median values in the post-test measurements.

For postoperative care knowledge, the median score remained at 5.00; however, the interquartile range narrowed from 3.00–5.00 to 5.00–5.00, indicating that all participants achieved the maximum score following the intervention (Z = −3.384, *p* < 0.001, r = 0.62).

In terms of feeding knowledge, the median score increased from 3.00 (IQR: 3.00–4.00) to 5.00 (IQR: 5.00–5.00), demonstrating substantial improvement after the intervention (Z = −4.451, *p* < 0.001, r = 0.81).

Complication monitoring knowledge had the lowest baseline scores among all subdimensions. The median score increased from 1.00 (IQR: 0.00–2.25) before the intervention to 5.00 (IQR: 5.00–5.00) after the intervention (Z = −4.329, *p* < 0.001, r = 0.79).

For the total knowledge score, the mean pre-test score was 8.60 ± 3.76, with a median of 8.00 (IQR: 6.00–10.25). In the post-test, the mean increased to 14.67 ± 0.92, and the median reached 15.00 (IQR: 15.00–15.00), indicating that nearly all participants achieved the maximum score.

Overall, statistically significant improvements were observed in all knowledge domains and in total knowledge scores. The clustering of post-test scores at the upper limit suggests a possible ceiling effect and reduced variability among participants following the educational intervention.

To evaluate the effect of parental education level on changes in knowledge regarding postoperative care, feeding, and complication monitoring after cleft lip and/or palate surgery, delta (change) scores were compared using the Kruskal–Wallis test (Table 4). The results indicated that differences in total knowledge gain and subdimension change scores were not statistically significant across parental education levels (*p* > 0.05).

In terms of total knowledge gain, the mean rank was higher among mothers with primary education (19.36) compared to those with high school education (14.33). Similarly, for knowledge gain, mothers with primary education demonstrated a higher mean rank (19.93). Although these differences did not reach statistical significance, it is noteworthy that the *p*-value for feeding knowledge gain was close to the significance threshold (*p* = 0.096).

These findings suggest that the educational intervention was effective regardless of parental education level. However, there appears to be a tendency toward greater knowledge gains among mothers with lower levels of education.

To determine in which knowledge domains the educational intervention provided greater gains, delta (change) scores for postoperative care knowledge, feeding knowledge, complication monitoring knowledge, and total knowledge score were compared using the Friedman test. The analysis revealed a statistically significant difference in the level of improvement across knowledge domains (χ^2^(3) = 56.30, *p* < 0.001).

Examination of mean rank values indicated that the highest gain was observed in the total knowledge score (mean rank = 3.70), suggesting that the educational program had a strong overall impact on improving knowledge. Among the subdimensions, the greatest improvement was observed in complication monitoring knowledge (mean rank = 2.65), followed by feeding knowledge (mean rank = 1.87) and postoperative care knowledge (mean rank = 1.78) (Table 5).

**Figure 3:**
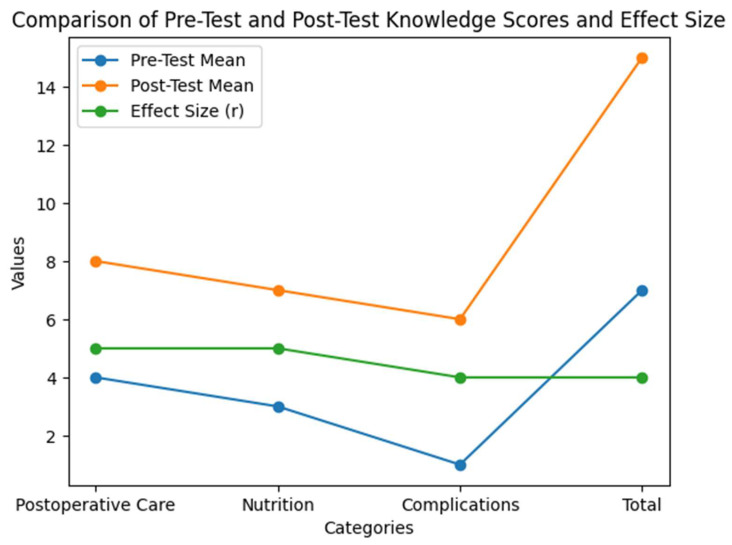
Comparison of participants’ pre-test and post-test knowledge levels and effect sizes.

Although significant improvements were also observed in feeding and postoperative care knowledge, the magnitude of gain in these domains was relatively lower compared to complication monitoring knowledge. This may be attributed to lower baseline knowledge levels (Figure 3) in complication monitoring, allowing the educational intervention to address a greater knowledge gap in this area.

Overall, the Friedman test results indicate that the educational intervention was effective across all knowledge domains, with a preliminary suggestion of improvement in complication monitoring knowledge and a substantial overall increase in total knowledge levels.

## 4. Discussion

This pilot study evaluated changes in mothers’ knowledge of postoperative care after they watched an AI-supported video-based educational material prepared for families of children undergoing cleft lip and/or palate surgery [16,17,18,19,20]. Knowledge scores increased in all domains, including postoperative care, feeding, and complication monitoring. The total knowledge score also improved after the intervention. Although these findings are encouraging, they should be interpreted with caution because the study used a single-group pre–post design and assessed knowledge immediately after the educational session. Therefore, the results mainly reflect short-term knowledge gain rather than sustained learning or direct changes in caregiving practice.

The greatest improvement was observed in complication monitoring, which was also the domain with the lowest baseline score. This finding is clinically relevant. After cleft lip and/or palate surgery, mothers are expected to recognize warning signs, monitor feeding and wound healing, maintain oral hygiene, and seek timely professional support when needed [3,4,8,9,10,11]. These responsibilities can be difficult to manage, particularly when mothers receive large amounts of information during a stressful postoperative period. The marked improvement in complication monitoring suggests that visual and narrative educational formats may be useful for explaining risk-related information in a clearer and more memorable way. Instructions that may seem abstract when delivered verbally can become easier to understand when they are shown step by step through visual examples.

The findings also underline the importance of the educational format itself. Verbal explanations given during routine clinical care are essential, but they may be limited by time pressure, maternal anxiety, fatigue, and the amount of information that needs to be remembered. A video-based format offers mothers the opportunity to pause, replay, and revisit the same content after discharge. This feature may be particularly valuable in postoperative home care, where uncertainty often arises after families leave the hospital. In this sense, the video did not simply deliver information; it provided mothers with a repeatable source of guidance that could reinforce the instructions given by healthcare professionals. This is consistent with previous work suggesting that the way health information is delivered can influence understanding, recall, and confidence in care [12,13].

The role of artificial intelligence in the present study should also be discussed carefully. Recent studies have shown that AI-based tools may contribute to patient education by providing structured and accessible health information in different clinical fields, including plastic surgery and orthopedic trauma care [21,22]. However, these studies also emphasize the need for professional oversight, clinical accuracy, and careful review of AI-supported content. In the present study, AI was not used to generate individualized recommendations, provide real-time feedback, or guide clinical decision-making. Its role was limited to the production of the educational material, including avatar creation, voice synthesis, and visual design. The educational content itself was prepared by the researchers and reviewed for clinical appropriateness. For this reason, the intervention should be understood as an AI-supported video-based educational material, not as an autonomous AI-driven education or decision-support system.

The findings should also be considered within the broader caregiving experience of mothers of children with cleft lip and/or palate. Postoperative education is not only a matter of transferring information. Mothers may also experience fear, uncertainty, and low confidence when they are expected to care for their child after surgery. Çınar and Koç reported that structured nursing care provided to mothers of infants with cleft lip and palate improved maternal attachment, parental self-efficacy, coping skills, and maternal adaptation [23]. Similarly, Çınar et al. described having an infant with cleft lip and/or palate as an unexpected and emotionally challenging experience for mothers, highlighting the importance of professional, family, and social support [24]. These findings suggest that educational materials are most meaningful when they are integrated into a family-centered care approach that addresses both informational and emotional needs.

It should also be noted that the observed knowledge gains cannot be attributed solely to the video intervention. Mothers who stayed longer in the hospital may have had more opportunities to ask questions, observe care practices, and receive repeated explanations from nurses and other healthcare professionals. Therefore, the AI-supported video-based material may have acted as one part of a wider learning process rather than as the only source of improvement. This interpretation is important because it places the video within the real clinical context in which mothers learn: through verbal explanations, observation, repeated contact with professionals, and access to supportive materials.

Overall, the results suggest that AI-supported video-based education may be a useful adjunct to postoperative family education after cleft lip and/or palate surgery. Its main value lies in reinforcing key care messages in a consistent and accessible format, especially after discharge. However, it should not be viewed as a replacement for individualized nursing education or professional counselling. Future randomized controlled studies with larger samples, longer follow-up periods, and comparison groups receiving routine verbal education, written materials, or non-AI video education are needed to determine whether this approach improves long-term knowledge retention, caregiving practices, and clinical outcomes.

### Limitations

Several limitations bear on the interpretation of these findings. The single-center design and small sample restrict generalizability, and the absence of a control group means that the observed knowledge gains cannot be attributed to the intervention alone. Repeated testing, routine verbal instruction, interaction with clinical staff, and prior caregiving experience are all plausible contributors to the post-test scores.

The measurement instrument introduces further uncertainty. The questionnaire was developed specifically for this pilot study and, while content validity was supported through expert review and pre-test internal consistency was satisfactory, construct validity and test–retest reliability were not assessed. The clustering of post-test scores near the ceiling also suggests that the instrument may have lacked the sensitivity to detect meaningful variation in maternal knowledge after the intervention.

Finally, because measurement was confined to the immediate post-intervention period, nothing can be said about long-term retention, actual caregiving behavior at home, or clinical outcomes after discharge. Whether AI-supported video education offers advantages over conventional verbal instruction, written materials, or non-AI video formats remains an open question—one that controlled studies with larger samples and longer follow-up are better positioned to answer.

## 5. Conclusions

This pilot study suggests that AI-supported video-based education may be a feasible and supportive tool for improving mothers’ short-term knowledge of postoperative care after cleft lip and/or palate surgery. However, because of the single-group pre–post design, small sample size, and absence of a control group, these findings should be interpreted cautiously. Larger randomized controlled studies with long-term follow-up are needed to determine whether this approach provides benefits beyond conventional verbal instruction, written materials, or non-AI video-based education.

## Figures and Tables

**Table 1:** Sociodemographic Characteristics of Mothers and Children (N = 30).

Variable	n	%
**Gender of Children**		
Male	13	43.3
Female	17	56.7
**Age Group of Children**		
0–3 years	17	56.7
3–11 years	6	20.0
11 years and older	7	23.3
**Parental Education Level**		
Primary Education	7	23.3
High School	23	76.7

Note: The values presented in the table are expressed as frequency (n) and percentage (%).

**Table 2:** Distribution of Children’s Clinical Characteristics (N = 30).

Variable	n	%
**Type of Diagnosis**		
Cleft lip	15	50.0
Cleft palate	14	46.7
Cleft lip and palate	1	3.3
**Surgical Procedure Performed**		
Cleft lip surgery	14	46.7
Cleft palate surgery	14	46.7
Cleft lip and palate surgery	2	6.7
**Chronic Disease**		
None	30	100.0
**Family History**		
None	30	100.0

Note: Values in the table are presented as frequency (n) and percentage (%).

**Table 3:** Comparison of Pre-test and Post-test Knowledge Scores Regarding Postoperative Care After Cleft Lip and/or Palate Surgery.

Variable	Pre-Test Median (IQR)	Post Test Median (IQR)	Z	*p*	r
Postoperative care knowledge	5.00 (3.00–5.00)	5.00 (5.00–5.00)	−3.384	<0.001	0.62
Nutrition knowledge	3.00 (3.00–4.00)	5.00 (5.00–5.00)	−4.451	<0.001	0.81
Complication monitoring knowledge	1.00 (0.00–2.25)	5.00 (5.00–5.00)	−4.329	<0.001	0.79
Total knowledge score	8.00 (6.00–10.25)	15.00 (15.00–15.00)	−4.297	<0.001	0.78

Note. IQR = Interquartile range (25th–75th percentile); Z = Wilcoxon Signed-Rank Test statistic; r = effect size (r = |Z|/√N). Negative Z values indicate that post-test scores were higher than pre-test scores.

**Table 4:** Comparison of Knowledge Level Change (Delta) Scores According to Parental Education Level (N = 30).

Variable	Education Level	n	Mean Rank	H	df	*p*
Total knowledge increase	Primary Education	7	19.36	1.79	1	0.181
	High School	23	14.33			
Postoperative care knowledge increase	Primary Education	7	16.43	0.12	1	0.726
	High School	23	15.22			
Nutrition knowledge increase	Primary Education	7	19.93	2.77	1	0.096
	High School	23	14.15			
Complication monitoring knowledge increase	Primary Education	7	18.36	1.01	1	0.314
	High School	23	14.63			

Note: Change (delta) scores were calculated from the difference between post-test and pre-test scores. The Kruskal–Wallis test was used to compare groups.

**Table 5:** Comparison of Change (Delta) Scores Across Knowledge Domains Using the Friedman Test (N = 30).

Variable	Mean Rank
Total knowledge increase (Δ Total)	3.70
Complication monitoring knowledge increase (Δ Complication)	2.65
Nutrition knowledge increase (Δ Nutrition)	1.87
Postoperative care knowledge increase (Δ Care)	1.78

Note: Change (delta) scores were calculated from the difference between post-test and pre-test scores. Gain levels across knowledge domains were compared using the Friedman test χ^2^(3) = 56.30, *p* < 0.001.

## Data Availability

The data that support the findings of this study are not publicly available due to privacy and ethical restrictions. However, anonymized data may be made available from the corresponding author upon reasonable request and with the permission of the relevant ethics committee (Supplementary File S3).

## Supplementary Materials

The following supporting information can be downloaded at: https://www.mdpi.com/article/10.3390/healthcare14142182/s1, Supplementary File S1: AI-supported video-based education; Supplementary File S2: TREND checklist; Supplementary File S3: De-identified SPSS dataset. Ref. [25] was cited in Supplementary Materials.

## Abbreviations

The following abbreviations are used in this manuscript:

CL/P	Cleft Lip and Palate
CP	Cleft Palate
