# Supplementary material for: Effectiveness of a Family Education Intervention Using an AI-Supported Video in Postoperative Care of Children with Cleft Lip and Palate: A Pilot Pre–Post Study
Source: Healthcare (Basel). 2026 Jul 20;14(14):2182. doi: 10.3390/healthcare14142182 (PMC13410092; doi:10.3390/healthcare14142182)
Supplement: Supplementary file 1 [file healthcare-14-02182-s001.zip › File S2. TREND Checklist.pdf]

TREND Statement Checklist

Section / Topic	Item No	TREND Item (Descriptor)	Reported?	Page No
Title and Abstract	**1**	Information on how units were allocated to interventions.<br>Structured abstract recommended.<br>Information on target population or study sample.	**Yes**	1-2
Introduction (Background)	**2**	Scientific background and explanation of rationale.<br>Theories used in designing behavioral interventions.	**Yes**	2-3
Methods Participants	**3**	Eligibility criteria for participants, including criteria at different levels.<br>Method of recruitment/sampling.<br>Recruitment setting.<br>Settings and locations where data were collected.	**Yes**	3-4
Methods Interventions	**4**	Details of the intervention for each study condition: content, delivery method, unit of delivery, deliverer, setting, exposure quantity/duration, time span, compliance-enhancing activities.	**Yes**	3, 5-6
Methods Objectives	**5**	Specific objectives and hypotheses.	**Yes**	3
Methods Outcomes	**6**	Clearly defined primary/secondary outcome measures.<br>Data collection and measurement-quality methods.<br>Validity/reliability information for the instrument used.	**Yes**	5-6, 9
Methods Sample Size	**7**	How sample size was determined; interim analyses/stopping rules, if applicable.	**Yes**	3-4
Methods Assignment Method	**8**	Unit of assignment; method used to assign units to study conditions; steps taken to minimize bias (e.g., matching).	**Not Applicable**	3, 4
Methods Blinding	**9**	Whether participants, those administering the intervention, and those assessing outcomes were blinded to study condition.	**No**	–
Methods Unit of Analysis	**10**	Description of the smallest unit analyzed; if different from the unit of assignment, the method used to account for this.	**Yes**	7-9

Section / Topic	Item No	TREND Item (Descriptor)	Reported?	Page No
Methods<br>Statistical Methods	**11**	Statistical methods for comparing groups on the primary outcome(s), including correlated data.<br>Methods for additional analyses.<br>Methods for imputing missing data.<br>Statistical software used.	**Yes**	7
Results<br>Participant Flow	**12**	Flow of participants through enrollment, assignment, exposure, follow-up, and analysis (flow diagram recommended).<br>Description of protocol deviations.	**Yes**	4
Results<br>Recruitment	**13**	Dates defining the periods of recruitment and follow-up.	**Yes**	3
Results<br>Baseline Data	**14**	Baseline demographic/clinical characteristics.<br>Disease-specific baseline characteristics.<br>Comparison of completers vs. those lost to follow-up.<br>Comparison of study population to target population.	**Yes**	8
Results<br>Baseline Equivalence	**15**	Data on baseline group equivalence and statistical methods used to control for baseline differences.	**Not Applicable**	–
Results<br>Numbers Analyzed	**16**	Number of participants (denominator) in each analysis.<br>Whether an "intention to treat" strategy was used, or how non-compliers were handled.	**Yes**	3-4, 8
Results<br>Outcomes and Estimation	**17**	Summary of results and effect-size estimate (with precision) for each outcome by study condition.<br>Inclusion of null/negative findings.<br>Results of tests of pre-specified causal pathways.	**Yes**	9-11
Results<br>Ancillary Analyses	**18**	Summary of other analyses performed, including subgroup or restricted analyses, indicating whether pre-specified or exploratory.	**Yes**	10
Results<br>Adverse Events	**19**	Summary of all important adverse events or unintended effects in each study condition.	**No**	–
Discussion<br>Interpretation	**20**	Interpretation of results considering hypotheses, sources of bias, imprecision, and other limitations.<br>Discussion of causal pathways.<br>Success of/barriers to implementation, fidelity.<br>Research/programmatic/policy implications.	**Yes**	11-13

Section / Topic	Item No	TREND Item (Descriptor)	Reported?	Page No
Discussion Generalizability	**21**	Generalizability (external validity) considering study population, intervention characteristics, follow-up length, incentives, compliance rates, specific sites/settings, and other contextual issues.	**Yes**	13
Discussion Overall Evidence	**22**	General interpretation of results in the context of current evidence and current theory.	**Yes**	11-13

From: Des Jarlais, D. C., Lyles, C., Crepaz, N., & the Trend Group (2004). Improving the reporting quality of nonrandomized evaluations of behavioral and public health interventions: The TREND statement. American Journal of Public Health, 94, 361-366. For more information, visit: http://www.cdc.gov/trendstatement/
